# A PPE detection method based on the advanced YOLOv11 architecture with a lightweight refinement block system

**DOI:** 10.1371/journal.pone.0361911

**Published:** 2026-11-10

**Authors:** Alibek Barlybayev, Nurzada Amangeldy, Bijeesh Kozhikkodan Veettil, Bekbolat Kurmetbek, Akmaral Kassymova, Ramazan Sadvakassov

**Affiliations:** 1 Faculty of Information Technologies, L.N. Gumilyov Eurasian National University, Astana, Kazakhstan; 2 SignBridge LLP, Astana, Kazakhstan; 3 Laboratory of Ecology and Environmental Management, Science and Technology Advanced Institute, Van Lang University, Ho Chi Minh City, Vietnam; 4 Faculty of Applied Technology, Van Lang School of Technology, Van Lang University, Ho Chi Minh City, Vietnam; 5 Institute of Economics, Information Technologies and Professional Education, Zangir Khan West Kazakhstan Agrarian-Technical University, Uralsk, Kazakhstan; Autonomous University of Queretaro: Universidad Autonoma de Queretaro, MEXICO

## Abstract

Automated monitoring of personal protective equipment usage is crucial due to the high incidence of accidents in the construction industry, with over half attributed to the improper use of protective equipment. Existing deep learning models show limited performance in detecting small-scale objects and reduced accuracy under challenging visual conditions. To address these limitations, this study introduces YOLOv11s-R^3^, an enhanced version of the YOLOv11s architecture with a P2-focused refinement block. The proposed model extends the standard feature pyramid network to stride-4 resolution and applies a single lightweight C2f block on the P2 feature map, targeting fine-scale objects. The model was trained on the Color Helmet and Vest dataset and evaluated on its held-out test split and on the GDUT Hardhat Wearing Detection dataset, with all experiments repeated across five independent random seeds. On the CHV test split, YOLOv11s-R^3^ achieved a precision of 0.9103 and mAP@50 of 0.9032, compared to 0.9035 and 0.9011 for the YOLOv11s baseline. The model maintained an inference speed of approximately 5 ms per image (190 FPS on an NVIDIA Quadro RTX 6000 GPU). On the CHV test split, the observed mAP@0.5 gain was not statistically distinguishable from run-to-run variance (paired t-test, p = 0.369). In contrast, the improvement observed on GDUT-HWD was more consistent relative to run-to-run variance, providing the primary evidence that the benefit of the proposed refinement extends across datasets. Together with an inference speed of approximately 5 ms per image, these findings support the applicability of YOLOv11s-R^3^ to real-time PPE monitoring.

## Introduction

Occupational safety is a critically important issue for researchers and engineering practitioners worldwide, and the construction industry is considered one of the most dangerous industries, characterized by a high rate of accidents and fatalities [1,2]. Construction workers face a risk of injury that is twice as high as in other industries [1]. Wearing personal protective equipment (PPE) is a fundamental requirement for all employees to prevent workplace accidents [3,4]. The adoption of proactive safety measures, including the consistent use of PPE, has proven effective in reducing injury rates. Statistics emphasize the urgent need for such measures: more than half (56%) of accidents on construction sites are caused by the lack of PPE [4], and in 2020 in Taiwan, up to 71% of serious occupational injuries were caused by unsafe equipment, including a lack of PPE [2]..

Traditional methods of monitoring compliance with PPE requirements rely on manual inspection [4]; however, human supervision is time-consuming, expensive, and impractical when monitoring a large number of employees [5]. In this regard, there is an urgent need for automated and reliable monitoring systems.

In recent years, deep learning and machine learning have become transformative technologies that have substantially advanced the capabilities of computer vision. Object detection is a key aspect that is widely used in the context of security control and compliance with the standards for the use of PPE. An effective monitoring system should be capable of determining in real time whether employees comply with prescribed safety protocols [2,3].

Among the existing object detection algorithms, architectures based on the You Only Look Once (YOLO) concept are popular due to their high speed and accuracy [5–7]. YOLOv8, being one of the latest deep learning models [8], has demonstrated exceptional performance by successfully detecting protective helmets (0.969), vests (0.887), and gloves (0.857) [3].

Despite considerable progress, modern models face serious challenges, especially in complex real-world scenarios:

1. Detection of small objects: Detection efficiency is often reduced when working with small objects [9]. The pixels of small targets are very small, which can lead to objects overlapping in complex scenes, making it difficult to extract information [3,9].
2. Difficult environmental conditions (e.g., low light): The accuracy of PPE detection may decrease due to low light conditions and color variations [3]. While low-light detection, such as in tunnel construction sites, represents an important direction, it is not directly addressed by the datasets used in the present study and is therefore deferred to future work [9–11].

To date, there is a lack of research specifically aimed at developing suitable models for detecting small PPE objects in complex construction site conditions [9]. To address this limitation, this paper proposes YOLOv11s-R^3^, an extension of YOLOv11s in which the architectural modification is explicitly targeted at stride-4 resolution – the feature scale most relevant for small PPE objects. Rather than applying general-purpose attention mechanisms, the proposed refinement block operates directly on stride-4 feature maps, retaining the high-resolution spatial information that is critical for localizing small PPE objects.

The rest of the article is organized as follows: Section 2 provides an overview of the literature on modern PPE detection methods; Section 3 describes the architecture of the proposed improved YOLOv11 method, the architecture and operating principle of the proposed P2-focused refinement block, as well as the experimental setup and the data set used; Section 4 provides experimental results and a comparative analysis of key accuracy and performance metrics; Section 5 contains a discussion of the results obtained; finally, Section 6 sets out the main conclusions and directions for further research.

## Literature review

### The YOLO family of algorithms

The YOLO family of algorithms is a key focus in the development of computer vision systems for monitoring PPE, as these single-stage detectors provide high speed and accuracy in real time [12]. YOLOv3 was one of the early iterations that used the Darknet-53 architecture and was able to make predictions on three different scales (for example, 13×13, 26×26, 52×52 for a 416×416 entrance), which helped to detect objects of different sizes. YOLOv3 uses logistic regression and several independent logistic classifiers with the Sigmoid function to calculate the reliability of the class. This allows an object to potentially belong to several classes, which is important for complex scenes [1,2].

YOLOv4 incorporated substantial improvements, including the new CSPDarknet53 backbone. This version used optimization techniques known as “Bag-of-Freebies” and “Bag-of-Specials,” which increase accuracy with limited impact on speed. To augment the data, YOLOv4 uses the Mosaic method, which randomly combines four training images, which is effective for improving the detection of small objects. CIoU (Complete Intersection over Union) is used as a loss function for the regression of bounding boxes. Due to the large size and computational complexity of the YOLOv4 base model (for example, 235 MB), compression methods such as CLSlim were developed that combine channel trimming (L1 regularization) and layer trimming, which reduced the model parameters by 98.2% (up to 4.15 MB) and increased the output speed by 1.8 times [13].

YOLOv5 is a widely used version that has been translated into the PyTorch framework. YOLOv5 has five main variants (n, s, m, l, x). The fastest variant, YOLOv5n, showed the fastest detection rate – 70.4 FPS. The largest variant, YOLOv5x, demonstrates the best average accuracy (mAP) of 95.8% in the task of detecting helmets [14]. Improved versions, such as ME-YOLO (based on YOLOv5), use additional modules: C3_FFM for a complete fusion of semantic and localization information and the Up-Sampling Enhancement Module (USEM), including CARAFE, to preserve global features while increasing sampling [15].

YOLOv7 introduced a new architecture, E-ELAN (Extended Efficient Layer Aggregation Network), and uses the technique of reparametrization [16]. This model was recognized as one of the best in terms of achieving a balance between speed and accuracy. In PPE detection studies, YOLOv7 has been enhanced by incorporating a combined attention module comprising a CBAM (Convolutional Block Attention Module) and an SE (Squeeze-and-Excitation) block positioned directly in front of the detection layer. YOLOv8 switched to a decoupled head anchor-free architecture. However, early versions of YOLOv8 relied on DarkNet-53, which limited the capture of fine-grained features, especially for small and occluded objects [17]. YOLOv9 introduced Programmable Gradient Information (PGI) to improve gradient flow. The YOLOv9-e variant showed the best performance among the tested variants on the SH17 dataset, reaching a 70.9% mAP@50 [18]. YOLOv10 increased the output speed by eliminating the Non-Max Suppression (NMS) post-processing stage through the use of a consistent dual assignment strategy. This made it possible to reduce the number of trained parameters by 15% compared to YOLOv9-c with comparable performance [18].

YOLOv11 is one of the latest basic architectures focused on balancing efficiency and accuracy. The YOLOv11n variant uses deep convolution (DWConv) in the head module to reduce computational costs. Advanced variants such as SC-YOLO (based on YOLOv11n) use CSPDarknet and include Sophia, a scalable second–-order stochastic optimizer. Sophia uses Hessian estimates to provide curvature-aware updates, resulting in faster convergence with minimal additional cost (only 7% computational overhead) [19]. SC-YOLO showed a 9.03% improvement in detecting transparent objects (such as safety glasses). Another improved variant, GCB-YOLOv11, designed for complex mining environments, uses GSConv to improve feature extraction and reduce complexity, as well as Bi FPN to optimize the fusion of multiscale features. GCB-YOLOv11 achieved 93.6% mAP and 90.3 FPS [20,21].

The evolution of the YOLO family demonstrates a steady trend towards increasing accuracy while maintaining high processing speeds, which makes these models particularly suitable for PPE monitoring systems. Since YOLOv3, each subsequent version has made architectural improvements, from the introduction of CSPDarknet and Path Aggregation Network (PAN) in YOLOv4 to the anchor-free and decoupled head in YOLOv8, as well as PGI and GELAN in YOLOv9. The latest generations, YOLOv10 and YOLOv11, are focused on reducing computing costs and eliminating NMS postprocessing, while maintaining comparable accuracy. The SC-YOLO and GCB-YOLOv11 variants show that the modern YOLO architecture is capable of providing accurate and stable recognition of small objects such as hard hats, vests, and gloves in construction sites with high levels of visual noise. A brief comparison of the key generations of YOLO is presented in Table 1.

**Table 1:** Evolution of YOLO Architectures and Their Relevance for PPE Detection.

YOLO Version	Key Architectural Improvements	Relevance for PPE Detection
YOLOv3	Utilizes logistic regression and independent sigmoid classifiers instead of Softmax for class prediction. Detects objects at three different scales [1,2].	Used in multi-stage approaches to verify correct PPE usage in combination with OpenPose.
YOLOv4	Introduced the CSPDarknet53 architecture, Mosaic data augmentation, and an improved feature fusion mechanism [13].	Offers increased accuracy while maintaining inference speed. The CLSlim-YOLOv4 variant demonstrated a 98.2% parameter reduction with minimal mAP loss, which is critical for mobile applications.
YOLOv5	Implemented in the PyTorch framework. Model variants (n, s, m, l, x) differ in size and performance [14,15].	The YOLOv5x model achieved the highest performance (mAP 95.8%), while YOLOv5n provided the fastest inference speed (70.4 FPS), which is essential for real-time deployment. Serves as a foundation for advanced models (e.g., ME-YOLO, YOLO-EfficientNet).
YOLOv7	Introduced the E-ELAN (Extended Efficient Layer Aggregation Network) architecture [16].	Achieves record-breaking speed and accuracy, representing the state-of-the-art among real-time detectors.
YOLOv8	Follows a one-stage detection approach. The architecture consists of Backbone, Neck, and Prediction Head components. Employs a decoupled head and an anchor-free concept. Utilizes CIoU and DFL losses for bounding box regression. Available in variants ranging from YOLOv8n (fewer parameters, higher accuracy) to YOLOv8x [17].	The main limitation of YOLOv8 was its dependency on DarkNet-53, which restricted fine-grained feature extraction. The YOLOv8-x variant achieved an mAP@50 of 69.3% on the SH17 dataset.
YOLOv9	Introduced PGI and GELAN (Generic Extended Layer Aggregation Network) modules. Developed to overcome YOLOv8’s limitations [18].	Demonstrates performance comparable to YOLOv8-l while using 32% fewer trainable parameters. The YOLOv9-e variant achieved an mAP@50 of 70.9% on the SH17 dataset.
YOLOv10	Improves inference speed by removing the NMS post-processing step. Introduces a consistent dual assignment strategy, adding a one-to-one assignment head alongside the one-to-many head. During inference, only one head is used.	Achieves performance comparable to YOLOv9-c while reducing the number of trainable parameters by 15%. The YOLOv10 model encounters challenges in detecting small objects. The YOLOv10-x variant achieved an mAP@50 of 67.8% on the SH17 dataset.
YOLOv11	Serves as the foundation for new variants such as SC-YOLO [19] and GCB-YOLOv11 [20]. The Head section incorporates two depthwise convolutions (DWConv), reducing both the number of parameters and computational cost.	Represents the latest evolutionary stage, combining efficiency and precision essential for industrial PPE monitoring.

Thus, YOLOv11 serves as a solid foundation for further research and optimization of automated safety compliance control systems.

### Fundamental deep learning architectures

Automated monitoring of PPE using deep learning is a critically important area for reducing the high level of injuries and deaths in construction and ensuring the integrity of protective suits for medical personnel [22]. Computer vision systems solve the problems of manual control, which is time-consuming and error-prone [23]. The main tasks for deep learning models are not only the detection of PPE (helmets, vests, boots), but also the classification of unsafe behavior and verification of the integrity of equipment after contact with infected patients [22]. Successful deep learning systems must overcome the complexities associated with real-time operation, different body positions, and a strong class imbalance in the data.

To detect objects in construction, architectures such as Faster R-CNN and SSD MobileNet V2 are used [23,24]. Faster R-CNN, being a two-stage detector, is successfully used to detect three combinations of PPE (helmets, vests, boots), achieving an average accuracy of 70% [23]. However, to ensure real-time operation and deployment on edge devices (AI edge cameras), the lightweight single-stage SSD MobileNet V2 architecture is preferred [24]. This model, which has only 3.47 million parameters, is optimized using methods such as post-training quantization and pruning, which reduces the size of the model file (for example, from 17.5 MB to 4.4 MB) and ensures low latency (0.2 seconds on camera). Augmentation methods such as SSD random crop, horizontal reflection, and color modifications are used to increase the stability of the model to real conditions [24].

Deep learning models are also used to classify complex unsafe behavior (for example, “man stretches” or “man sticks his legs out”), which accounts for 80–90% of all construction site incidents. In such tasks, the Transfer Learning approach is actively used, in which pre-trained deep convolutional neural networks (CNN) are configured on a smaller target dataset. A comparative analysis showed that InceptionResnet_V2 (depth 572) demonstrates the best performance among the tested models (including VGG19 and Inception_V3), achieving an accuracy of 92.44% [25].

Checking the integrity of the protective suit of medical personnel after contact with infectious patients (for example, Ebola) is a critical task. The detection of contaminants such as bloodstains is characterized by a strong class imbalance, as clean areas greatly outnumber (for example, contaminated areas account for only 0.0006% of pixels in synthetic data). In such conditions, algorithms capable of managing this imbalance are needed, such as Logistic Regression, Support Vector Machine (SVM) and eXtreme Gradient Boosting (XGB). XGBoost achieved the highest F1 score (80.68%) on a physically emulated dataset but required a long execution time. In general, Logistic Regression was recognized as the most suitable algorithm because it provides the most balanced results in terms of F1 score and run time (te) when working with both datasets [22].

Deep learning models have proven effective in automated PPE monitoring, covering both object detection (Faster R-CNN, SSD MobileNet V2) for monitoring on construction sites, as well as complex classification of behavior and suit integrity. Key architectural solutions include the use of efficient lightweight networks (SSD MobileNet V2) for deployment on peripheral devices and Transfer Learning with models such as InceptionResnet_V2 to accurately classify unsafe activities. When verifying the integrity of suits, it is critically important to use the F1 score metric to evaluate performance in conditions of severe class imbalance, while choosing the optimal algorithm (for example, Logistic Regression) depends on a compromise between accuracy and execution speed.

### Hybrid and specialized Models

Specialized hybrid approaches have been developed to increase the robustness of YOLO models. The NST-YOLOv5 model is a hybrid that combines the YOLOv5 architecture with Neural Style Transfer (NST) technology. The main task of NST is to synthesize training data simulating extreme conditions (low light, fog, rain, sand dust), since collecting real extreme data is difficult. The NST-YOLOv5 is trained on synthesized images, while the NST module is not loaded after training, which allows the model to maintain the same output speed as the standard YOLOv5. Experiments have shown that NST-YOLOv5 substantially increases robustness: the increase in mAP05:95 was 0.141 on synthesized data and 0.083 on real extreme data, which confirms the superiority of NST over traditional image processing algorithms [26].

Other modifications focus on optimizing the internal structure, such as the GBSG-YOLOv8n model, designed for an industrial environment with a complex background and varying target sizes. It also uses the SimC2f structure, which includes SimAM, a simple, parameterless attention mechanism, for more efficient processing of signs of small goals. To achieve lightness, traditional convolutions in Backbone have been replaced with GhostConv. GBSG-YOLOv8n achieved a 90.8% mAP with a substantial reduction in computational complexity [27].

The RFA-YOLO framework (modified YOLOv4) is used to detect PPE on offshore drilling platforms. RFA-YOLO includes the Residual Feature Augmentation (RFA) module, which reduces the loss of effective information from high-level feature maps. After objects are detected, the coordinates of the bounding boxes are used to construct positional features that characterize the relative displacements between objects (man/helmet or man/work clothes). These features are then fed into the optimized Random Forest Classifier (RFC). This hybrid approach makes it possible to accurately identify abnormal wearing (for example, holding a helmet in your hands), achieving an overall accuracy of 93.1% on the ODPD dataset [28].

To solve the problem of misclassification of an ordinary hat as a protective helmet, a two-stage YOLO-EfficientNet method was developed [29]. A high-precision detector, such as the YOLOv5x, is used to detect only the human head. This simplifies a multi-class detection task to a single class detection task. The detected head image is cropped and fed into the EfficientNetB7 classification model. EfficientNetB7 classifies the image into three classes: “helmet” (helmet), “head” (head without protection), and “hat” (hat). Transfer Learning with a “frozen” convolutional basis is used to train the classifier. YOLO-EfficientNet showed a macro F1 score of 93.7% and a mAP of 91.4%, surpassing other F1-score models such as Faster R-CNN and RetinaNet. This demonstrates a more suitable speed for real-time use than that of two-stage detectors.

The Multi-YOLOv5s-Transformer model was proposed to improve the detection of PPE (including small objects such as gloves and boots). It includes the SC3T module in the final layer, which combines SPP (Spatial Pyramid Pooling) with a layer of Transformer encoder and Multi-Head Self-Attention (MSA) to collect global information about features. In addition, this model replaces the CIoU loss function with the SIoU loss, which introduces a direction loss, allowing the predicted frame to align faster with the true frame. YOLOv5s-Transformer-SIoU reached 0.829 mAP50, which is notably higher than that of the original YOLOv5s [30].

The considered hybrid and specialized solutions demonstrate a wide range of directions for the development of YOLO models – from the generation of synthetic data using NST to the integration of Transformer blocks, attention mechanisms and two-stage classification schemes. Despite the increase in accuracy, most approaches remain limited either by computational complexity or by loss of speed when working in real time. Furthermore, these methods do not fully address the challenge of detecting small-scale PPE objects under challenging visual conditions, which motivates the present study [31].

Research issues

The research is aimed at solving critical problems in the field of automated control over the use of PPE on construction sites, especially in difficult conditions. Based on the analysis of the subject area and the goals set out in the sources, the following research questions can be formulated:

1. How can the key limitations of modern deep learning models in PPE detection be overcome, including reduced detection efficiency for small objects, which motivates the use of stride-4 feature maps in the proposed architecture?
2. Will the integration of a P2-focused refinement block into the YOLOv11s architecture (YOLOv11s-R^3^) achieve a consistent balance between detection accuracy and real-time processing speed?
3. To what extent does the proposed refinement strategy generalize to unseen data while maintaining stable detection performance under identical experimental conditions?

## Methodology

### Data

The Color Helmet and Vest reference dataset was used to train and evaluate deep learning models designed to detect PPE in construction sites [1,32]. The Color Helmet and Vest (CHV) dataset developed by Wang et al., contains 1,330 annotated images, comprising a total of 9,209 feature instances. It is designed for the tasks of detecting personnel and elements of PPE on construction sites. The images were selected from GDUT Hardhat Wearing Detection dataset (GDUT-HWD) and SHWD open sources, annotated manually using the labelImg tool, and converted to a YOLO-compatible annotation format [32]. The dataset includes six key classes of objects: person, vest, blue helmet, red helmet, white helmet, and yellow helmet. This class distribution covers the main categories of PPE used on construction sites and provides comprehensive training for detection models [32].

The CHV dataset was formed taking into account the actual operating conditions of computer vision systems. All images are obtained from existing construction sites, which provides a high level of realism and visual noise [1]. The workers are captured in various poses (standing, sitting, leaning) and from different angles — front, side, top, and back. There are a large number of small-scale objects on the screen (with an area of less than 32^2^ pixels), which makes the detection task especially difficult when analyzing video streams from long distances. In addition, when forming a dataset, a relative balance between categories is ensured, which contributes to sustainable learning and correct evaluation of models [1,32].

For an objective assessment of the quality of the CHV image models, they were divided into three subsets: training, validation, and test samples [32]. The training sample is 80% of the total (approximately 1064 images), the validation sample is 10% (about 133 images), and the test sample is 10% (about 133 images). This distribution allows for representative training and subsequent validation of the model on unseen data. The division took into account the balance of classes to avoid bias towards frequently occurring categories and to ensure an adequate representation of all types of objects [33,34].

The GDUT-HWD, introduced by Wu et al., was used as a second benchmark to assess the generalizability of the proposed method [35]. The dataset contains 3,174 images with 18,893 annotated instances across five object classes: blue helmet, white helmet, yellow helmet, red helmet, and none (indicating a person not wearing a hardhat). The images were collected from construction sites and cover a wide range of conditions, including varying visual ranges, illumination levels, individual postures, and partial occlusions [35]. A notable characteristic of the dataset is the high proportion of small-scale instances (area $\leq 32^{2}$ pixels), which makes detection particularly challenging. Although GDUT-HWD served as one of the source collections for the CHV dataset, the two differ in class composition: GDUT-HWD does not include the *person* and *vest* classes present in CHV. Following the original partition [35], 1,587 images were used for training and 1,587 images for testing. Both the YOLOv11s baseline and the proposed YOLOv11s-R^3^ were trained on GDUT-HWD under the same hyperparameter settings and across five independent random seeds. Because GDUT-HWD served as one of the source collections for CHV, the CHV test split and the full GDUT-HWD image pool were verified to contain no overlapping images using perceptual hashing (pHash, Hamming distance threshold ≤8); no duplicate pairs were detected.

### Improved architecture

In this research, we introduce an improved version of the YOLOv11 model, called YOLOv11s-R^3^, aimed at enhancing the detection accuracy of small PPE items while keeping the capability for real-time inference. The foundational YOLOv11 framework incorporates the C3K2, SPPF (Spatial Pyramid Pooling – Fast), and C2PSA (Channel–Spatial Attention) modules [36], which offer robust feature extraction and fusion abilities. Our proposed refinement strategy augments this framework by introducing a P2-focused refinement path after the standard Feature Pyramid Network (FPN) top-down branch, enabling the detection of fine-scale objects at stride-4 resolution (Fig 1).

**Fig 1:**
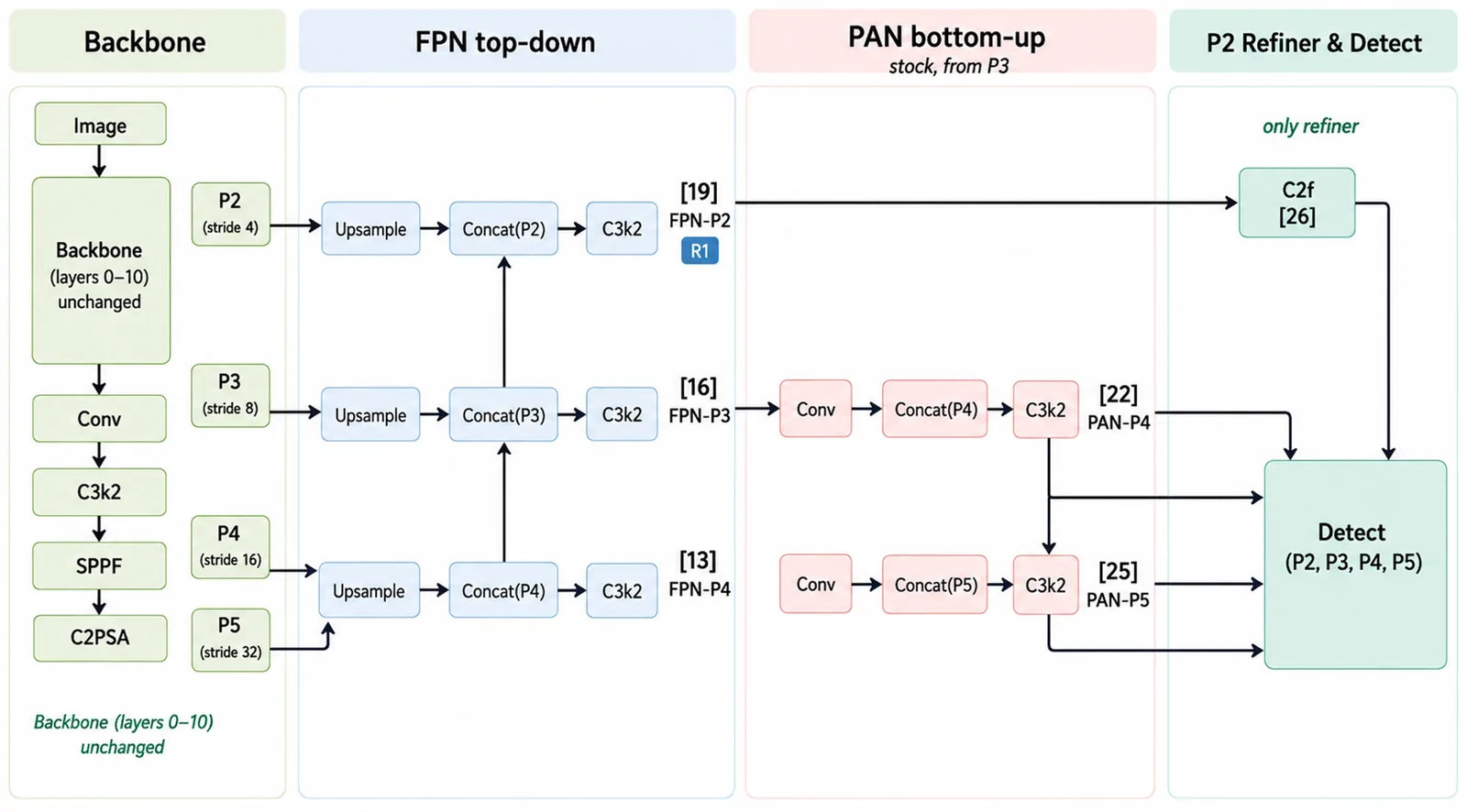
The proposed YOLOv11s-R^3^ architecture.

The overall design framework is composed of four main components:

1. *Backbone.* The feature extraction backbone sequentially applies Conv, C3k2, SPPF, and C2PSA modules to capture both low- and high-level spatial dependencies. The C2PSA attention block enhances the model’s ability to focus on semantically significant regions while suppressing background noise. The backbone is inherited unchanged from YOLOv11s.
2. *FPN.* The Feature Pyramid Network merges feature maps from multiple scales using upsampling, concatenation, and C3k2 blocks. The standard top-down path covers P5→P4→P3→P2, where the extension to P2 (stride-4) constitutes the R1 block (layer 19), recovering fine-grained spatial detail lost in deeper layers.
3. *PAN bottom-up.* The standard bottom-up path starts from P3 and progressively aggregates features up to P5 via Conv and C3k2 blocks (layers 22 and 25). The P2 scale is excluded from this path, leaving it unaffected by bottom-up processing.
4. *Refinement block and Detection head.* A single lightweight C2f block (layer 26) is applied exclusively on the P2 feature map prior to detection. The P3, P4, and P5 scales pass through the standard PAN bottom-up path without additional processing. The detection head operates on four scales: P2, P3, P4, and P5.

Each component plays a role in the multi-level feature refinement process. At the final stage, the detection head predicts bounding boxes, objectness scores, and class probabilities for each PPE category: *person, vest, blue helmet, red helmet, white helmet, and yellow helmet* (Fig 2). The P2 branch improves sensitivity to small-scale PPE items, such as gloves and glasses, while the standard PAN path preserves localization quality at larger scales.

**Fig 2:**
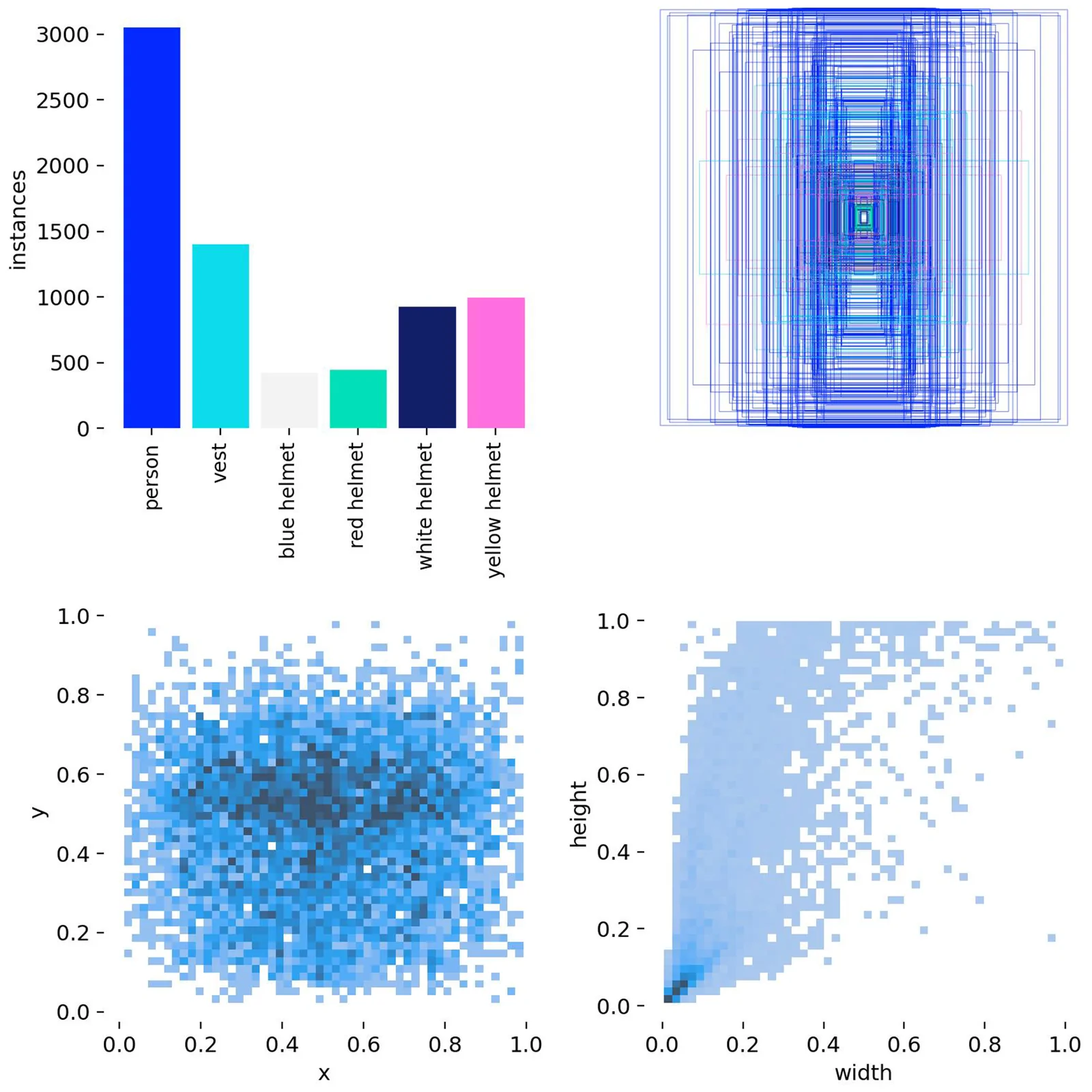
Distribution of annotation classes and characteristics in the CHV dataset.

The training objective is defined as:


$$\begin{array}{c} L=L_{\text{box}}+L_{\text{obj}}+L_{\text{cls}} \end{array}$$ (1)

where *L*_box_ represents the coordinate loss using the CIoU metric, *L*_obj_ corresponds to the binary cross-entropy of objectness, and *L*_cls_ denotes the multi-class cross-entropy for class prediction [36]. Optimization is performed using stochastic gradient descent (SGD) with a cosine learning rate decay schedule and gradient clipping to stabilize convergence.

Model training and evaluation were performed on the CHV dataset [32] and, to assess generalizability, on the GDUT-HWD dataset [35]. All images were resized to 640×640 px and annotated in YOLO-compatible format. To obtain statistically reliable results, all experiments were repeated across five independent random seeds and reported as mean ± standard deviation. Model performance was assessed using Precision, Recall, mAP@0.5, and mAP@0.5–0.95, which together capture complementary aspects of detection quality. Recall is particularly informative for small-scale instances, which are more likely to be missed than falsely detected.

The experiments were conducted on a server with four NVIDIA Quadro RTX 6000 GPUs (24 GB) running Ultralytics 8.3.54 / PyTorch 2.4.1 + CUDA 12.1. In the learning process, standard augmentations were used – mosaic, horizontal flip, brightness/contrast adjustment, and scale jittering – to increase the generalizing ability of the model. Both the YOLOv11s baseline and the proposed YOLOv11s-R^3^ were trained under the same conditions with the following parameters: batch size = 16, epochs = 200, learning rate = 0.01, momentum = 0.937, early stopping patience = 30, across five independent random seeds (0–4).

## Results

The proposed YOLOv11s-R^3^ achieved a mAP@50 of 0.9032±0.0058 and a mAP@50–95 of 0.5266±0.0064 on the CHV dataset, compared to 0.9011±0.0060 and 0.5409±0.0042 for the YOLOv11s baseline, averaged over five independent random seeds. A particularly notable improvement is observed in the recognition of helmets of different colors and small protective elements (see Fig 3a for the absolute confusion matrix and Fig 3b for the normalized one). An increase in the area under the PR curve (see Fig 4c) and an increase in F1 values (see Fig 4d) suggest improved overall precision and greater robustness to background noise with the P2-focused refinement.

**Fig 3:**
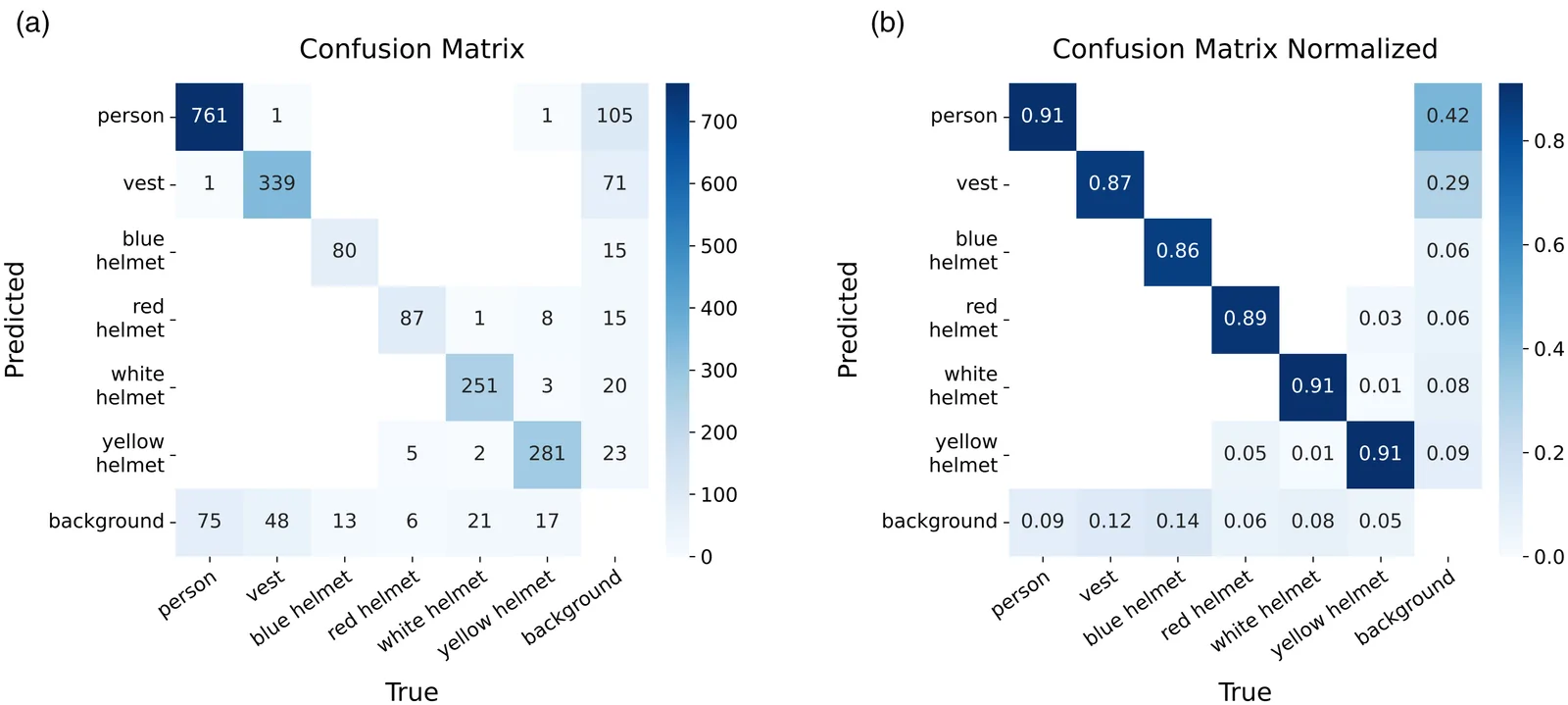
Confusion matrices: (a) absolute, (b) normalized for YOLOv11s-R^3^ (representative run, seed 2, selected as the median-performing seed by mAP@0.5–0.95 among the five runs).

**Fig 4:**
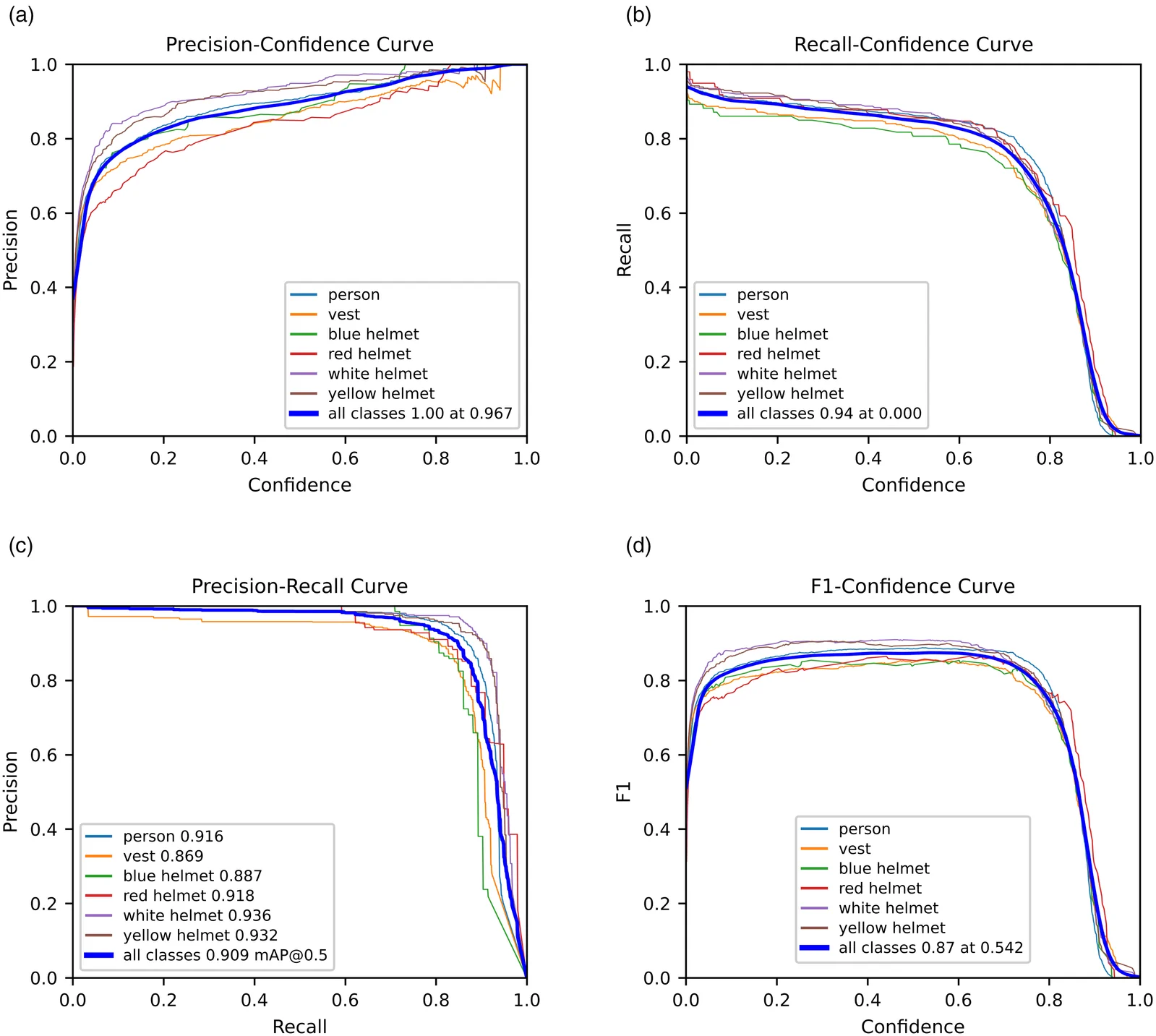
Precision, Recall, PR, and F1 curves for all PPE classes (representative run, seed 2, selected as the median-performing seed by mAP@0.5–0.95 among the five runs).

In the representative run (seed 2), the proposed model shows gains in precision for the white and red helmet classes, as illustrated by the Precision curve (see Fig 4a), and an improvement in recall for the yellow helmet class, as shown on the Recall graph (see Fig 4b); averaged over all five seeds, red helmet shows a small net regression in AP@0.5 (see Table 3).

These results are consistent with the conclusions of other studies on YOLOv11, where the addition of multi-level attention blocks and refinement modules increased accuracy with low computational costs [19,32]. The YOLOv11s-R^3^ model demonstrates resistance to visual noise and object overlap, which is especially important for use on construction sites with a dynamic environment and a heterogeneous background.

The average inference speed is about 5 ms/image, which corresponds to 190 FPS on the NVIDIA Quadro RTX 6000, confirming the suitability of the model for real-time PPE monitoring tasks. On the GDUT-HWD dataset, YOLOv11s-R^3^ achieved a mAP@50 of 0.9134±0.0037 compared to 0.9084±0.0028 for the baseline, demonstrating that the P2-focused refinement generalizes to a second benchmark with different class composition and scene conditions. Thus, the proposed YOLOv11s-R^3^ architecture provides improved PPE recognition quality through cascading feature refinement, which makes it promising for implementation in industrial video stream analysis systems and automatic safety monitoring.

## Discussion

The results obtained confirm the effectiveness of the proposed YOLOv11s-R^3^ architecture with a P2-focused refinement block. As shown in Table 2, on the CHV dataset YOLOv11s-R^3^ achieves higher precision (0.9103 vs. 0.9035), recall (0.8485 vs. 0.8411) and mAP@0.5 (0.9032 vs. 0.9011) compared to the YOLOv11s baseline, averaged over five independent random seeds. The baseline retains a higher mAP@0.5–0.95 (0.5409 vs. 0.5266), which indicates that the P2 refinement path primarily benefits detection at the standard IoU threshold rather than stricter localization criteria.

**Table 2:** Detection results on the CHV and GDUT-HWD datasets.

Dataset	Model	Precision	Recall	mAP@0.5	mAP@0.5–0.95
CHV	YOLOv11s	0.9035	0.8411	0.9011	**0.5409**
CHV	**YOLOv11s-R** ^**3**^	**0.9103**	**0.8485**	**0.9032**	0.5266
GDUT-HWD	**YOLOv11s-R** ^**3**^	**0.9050**	**0.8537**	**0.9134**	0.6232
GDUT-HWD	YOLOv11s	0.9026	0.8486	0.9084	**0.6248**

*Note:* Bold values indicate the best result per metric within each dataset.

A paired t-test on the per-seed mAP@0.5 results (n = 5) yields t=−1.011, *p* = 0.369 for the CHV dataset, indicating that the observed difference does not reach statistical significance at α=0.05 and should be interpreted with caution. On the GDUT-HWD dataset, the difference of 0.0050 (0.9134 vs. 0.9084) is more consistent relative to the observed variance (approximate t≈2.41), providing stronger evidence of generalization to an independent benchmark.

The reduction in mAP@0.5–0.95 on CHV (0.5266 for YOLOv11s-R^3^ versus 0.5409 for YOLOv11s) indicates that the improvement observed at the standard IoU threshold does not extend to stricter localization criteria. The class-wise results in Figs 3a–4d provide additional qualitative context on the detection behavior of YOLOv11s-R^3^ in the representative run (seed 2); for example, blue helmet exhibits the lowest normalized recall among the helmet classes (approximately 0.86) in that run.

Table 3 reports the per-class breakdown of both AP@0.5 and mAP@0.5–0.95, averaged over all five random seeds on the CHV validation split. For AP@0.5, person, vest, and white helmet show small but consistent gains for YOLOv11s-R^3^ (+0.0048, + 0.0084, and +0.0082, respectively), whereas blue helmet, red helmet, and yellow helmet show small regressions (−0.0022, −0.0074, and −0.0056), broadly consistent with the class-wise trends visible in Figs 4a–4b. For mAP@0.5–0.95, YOLOv11s-R^3^ regresses relative to the baseline in every class, but unevenly: red helmet shows by far the largest drop (−0.0302), roughly double the next-largest per-class regression (yellow helmet, −0.0165), while its AP@0.5 regression is comparatively modest (−0.0074).

**Table 3:** Per-class AP@0.5 and mAP@0.5–0.95 on the CHV validation set for YOLOv11s and YOLOv11s-R^3^, averaged over five independent random seeds (0–4).

Class	AP@0.5		mAP@0.5–0.95	
	YOLOv11s	YOLOv11s-R^3^	YOLOv11s	YOLOv11s-R^3^
Person	0.9120 ± 0.0060	**0.9168 ± 0.0040**	**0.5360 ± 0.0059**	0.5271 ± 0.0056
Vest	0.8692 ± 0.0078	**0.8776 ± 0.0061**	**0.5029 ± 0.0059**	0.4955 ± 0.0072
Blue helmet	**0.8696 ± 0.0190**	0.8674 ± 0.0145	**0.5015 ± 0.0101**	0.4894 ± 0.0100
Red helmet	**0.9134 ± 0.0227**	0.9060 ± 0.0149	**0.5280 ± 0.0136**	0.4978 ± 0.0052
White helmet	0.9290 ± 0.0064	**0.9372 ± 0.0084**	**0.5527 ± 0.0067**	0.5369 ± 0.0071
Yellow helmet	**0.9268 ± 0.0065**	0.9212 ± 0.0130	**0.5199 ± 0.0030**	0.5034 ± 0.0061
**Mean (all classes)**	0.9032	**0.9042**	**0.5235**	0.5083

*Note:* Values are mean ± standard deviation across five seeds, computed on the CHV validation split (see Discussion for comparison with the test-split results in Table 2). Bold indicates the higher value per class and metric.

This gap between a small AP@0.5 change and a large mAP@0.5–0.95 change for the same class suggests that red helmet is detected about as reliably as before, but with less precise bounding-box localization under stricter IoU thresholds – a class-specific effect rather than a uniform one across helmet categories. A reduction in aggregate mAP@0.5–0.95, though considerably smaller in magnitude, is also observed on GDUT-HWD (0.6232 versus 0.6248).

The improvement in precision and recall is attributed to the P2-focused refinement path, which extends the standard FPN top-down branch to stride-4 resolution and applies a single lightweight C2f block on the P2 feature map prior to detection. This design specifically targets small-scale PPE objects such as gloves and glasses, where fine-grained spatial detail is critical. The P3, P4, and P5 scales pass through the standard PAN path without additional processing, which preserves localization quality at larger scales.

From the point of view of computational efficiency, the number of YOLOv11s-R^3^ parameters increases by less than 8% relative to YOLOv11s, which is substantially less than in the cases of SC-YOLO or GCB-YOLOv11, where the parameter growth exceeds 25–30% [19,20]. The average inference speed remains approximately 5 ms/image (190 FPS on the NVIDIA Quadro RTX 6000), confirming suitability for real-time monitoring.

The experimental results show modest improvements in precision, recall, and mAP@0.5 over the YOLOv11s baseline on both datasets while maintaining real-time inference speed. However, the CHV mAP@0.5 gain was not statistically distinguishable from run-to-run variance, whereas the more consistent improvement observed on GDUT-HWD provides stronger evidence that the benefit of the P2-focused refinement extends across datasets.

## Conclusions

The proposed YOLOv11s-R^3^ extends YOLOv11s with a P2-focused refinement block that targets stride-4 features critical for small-scale PPE detection, while leaving the standard PAN path unchanged. The P2-focused refinement path extends the standard FPN top-down branch to stride-4 resolution and applies a single lightweight C2f block on the P2 feature map prior to detection. This design is architecturally oriented towards fine-scale object detection, targeting stride-4 features that correspond to small PPE items such as glasses or gloves. The P3, P4, and P5 scales pass through the standard PAN path without additional processing, preserving localization quality at larger scales.

The proposed architecture achieves a consistent balance between detection accuracy and real-time processing speed. On the CHV dataset, YOLOv11s-R^3^ achieved a mAP@50 of 0.9032 and precision of 0.9103, compared to 0.9011 and 0.9035 for the YOLOv11s baseline, averaged over five independent random seeds. The baseline retains a higher mAP@0.5–0.95 (0.5409 vs. 0.5266), indicating that the P2 refinement primarily benefits detection at the standard IoU threshold. The average inference speed is approximately 5 ms/image (~190 FPS on an NVIDIA Quadro RTX 6000 GPU), confirming suitability for real-time monitoring.

A paired t-test on the per-seed mAP@0.5 results showed that the observed difference was not statistically significant (p = 0.369), indicating that the CHV gain was not statistically distinguishable from run-to-run variance, while the more consistent improvement observed on GDUT-HWD provides the primary evidence of generalization across datasets.

### Directions for further research

Several limitations should be considered when interpreting these results. The improvement in mAP@0.5 on the CHV test split was not statistically distinguishable from run-to-run variance (paired t-test, p = 0.369), so the primary evidence for the benefit of the P2-focused refinement comes from the GDUT-HWD cross-dataset evaluation rather than the CHV result in isolation. YOLOv11s-R^3^ also shows a reduction in mAP@0.5–0.95 relative to the baseline on both datasets, indicating that the refinement does not uniformly improve localization quality under stricter IoU criteria. Evaluation was restricted to two datasets sharing broadly similar visual conditions (construction sites, daytime lighting), and GDUT-HWD does not include the person and vest classes present in CHV, which limits direct class-for-class comparison across datasets. Inference speed was measured on a workstation-class GPU (NVIDIA Quadro RTX 6000) rather than on edge or embedded hardware, the deployment context that motivates real-time PPE monitoring.

Motivated by these limitations, future work will focus on size-stratified evaluation to verify whether the P2 branch provides measurable gains specifically on small-scale PPE objects, and on identifying why the mAP@0.5–0.95 regression is concentrated in the red helmet class (Table 3) rather than distributed evenly across helmet categories. Additional directions include validation on dedicated low-light and indoor industrial datasets, adaptation to other base architectures, and deployment and latency benchmarking on edge devices for on-site real-time monitoring.
